# Psychometric evaluation of the Depression Anxiety Stress Scale 8 among women with chronic non-cancer pelvic pain

**DOI:** 10.1038/s41598-022-15005-z

**Published:** 2022-11-30

**Authors:** Amira Mohammed Ali, Amin Omar Hendawy, Rasmieh Al-Amer, Ghada Shahrour, Esraa M. Ali, Abdulmajeed A. Alkhamees, Nashwa Ibrahim, Sahar Mansour Taha Lamadah, Afaf Hassan Ahmed

**Affiliations:** 1 Department of Psychiatric Nursing and Mental Health, Faculty of Nursing, Alexandria University, Smouha, Alexandria, 21527 Egypt; 2 Department of Biological Production, Tokyo University of Agriculture and Technology, Fuchu, Tokyo 183-8538 Japan; 3 Department of Animal and Poultry Production, Faculty of Agriculture, Damanhour University, Damanhour, 22516 Egypt; 4 Faculty of Nursing, Isra University, Amman, Jordan; 5 Western Sydney University|School of Nursing and Midwifery, Penrith, NSW 2751 Australia; 6 Jordan University of Science and Technology, Faculty of Nursing, Irbid, 3030 Jordan; 7 Department of Basic and Educational Sciences, Faculty of Education for Early Childhood, Alexandria University, Mostafa Kamel, Alexandria, 21646 Egypt; 8 Department of Medicine, Unayzah College of Medicine and Medical Sciences, Qassim University, Unayzah, Al Qassim Saudi Arabia; 9 Psychiatric Nursing and Mental Health Department, Faculty of Nursing, Mansoura University, Mansoura, 30016 Egypt; 10 Department of Obstetrics and Gynecologic Nursing, Faculty of Nursing, Alexandria University, Alexandria, 21527 Egypt

**Keywords:** Psychology, Health care, Risk factors, Signs and symptoms

## Abstract

Psychiatric comorbidity and abusive experiences in chronic pelvic pain (CPP) conditions may prolong disease course. This study investigated the psychometrics of the Depression Anxiety Stress Scale 8 (DASS-8) among women with CPP (N = 214, mean age = 33.3 ± 12.4 years). The DASS-8 expressed excellent fit, invariance across age groups and menopausal status, good know-group validity (differentiating women with psychiatric comorbidity from those without comorbidity: U = 2018.0, p = 0.001), discriminant validity (HTMT ratios < 0.85), excellent reliability (alpha = 0.90), adequate predictive and convergent validity indicated by strong correlation with the DASS-21 (r = 0.94) and high values of item-total correlations (r = 0.884 to 0.893). In two-step cluster analysis, the DASS-8 classified women into low- and high-distress clusters (n = 141 and 73), with significantly higher levels of distress, pain severity and duration, and physical symptoms in cluster 2. The DASS-8 positively correlated with pain severity/duration, subjective symptoms of depression/anxiety, experiences of sexual assault, fatigue, headache severity, and collateral physical symptoms (e.g., dizziness, bloating, fatigue etc.) at the same level expressed by the parent scale and the DASS-12, or even greater. Accordingly, distress may represent a target for early identification of psychiatric comorbidity, CPP severity, experiences of sexual assault, and collateral physical complaints. Therefore, the DASS-8 is a useful brief measure, which may detect mental distress symptoms among women with CPP.

## Introduction

Pain in the pelvis unrelated to cancer, intercourse, or menstruation, which is experienced daily and persists for at least three consecutive months is known as chronic pelvic pain (CPP). CPP is commonly experienced by up to 22% of women^1–3^. It can be idiopathic or due to numerous urological, bowel-related, and gynecological reasons^3,4^. Because non-cancer CPP conditions in women worsen over time, especially during the peak reproductive years, fluctuations in ovarian hormones are suggested to be key modulators in the pathologies underlying CPP such as endometriosis and irritable bowel syndrome^1,5,6^.

Differences in CPP experience and pathology are reported between reproductive age women and peri/postmenopausal women^4^. Conditions entailing reduced levels of feminine hormones (e.g., menopause and during menses) are associated with increased sensitivity to visceral and pelvic pain^5^. In addition, ovarian collapse and subsequent reduction in estrogen during menopause is associated with numerous vegetative (e.g., hot flushes), physical (e.g., fatigue and back pain), urogenital/sexual (e.g., dyspareunia and urinary incontinence), cognitive (e.g., memory problems), and mental symptoms (e.g., depression and anxiety), which may aggravate pain sensitivity in midlife women and endanger their mental wellbeing^7^. In fact, CPP women within the age range of 25 to 35 years are reported to express less anxiety symptoms than their older counterparts^8^.

The annual healthcare expenditure of CPP is enormous, exceeding 6.5 billion dollars in Australia^2^. In addition, CPP alters women’s quality of life, reproductive capacity, social relations, work performance, and sexuality. As a result, CPP women experience high levels of distress, depression, and anxiety^9^. Depression, anxiety, and mixed anxiety depression disorder (MADD) prevail in 63%, 66%, and 54% of women experiencing CPP compared with 38%, 49%, and 28% of CPP-free women^8^. The etiopathogenesis of depression and anxiety in chronic pain is evoked by a complex mechanism through which pain signaling interferes with the pathophysiological and neurophysiological signaling, which induces mood dysfunction^10,11^. Moreover, chronic persistent rather than intermittent pain may exacerbate depressive and anxious manifestations by promoting constant patterns of cognitive biases (e.g., pain catastrophism), sleep disturbance, emotional dysregulation, behavioral inactivation, and loneliness^12,13^.

Exposure to different forms of abuse during early stages of life is associated with the development and persistence of numerous physical, emotional, mental, and sexual dysfunctions during adulthood^14^. Moreover, women in different parts of the world witness the highest exposure to different forms of abusive behavior against adults, along with numerous grave consequences^15^. Physical and sexual abuse represent a major risk factor for CPP^3,8^. Given the traumatic origins of CPP and the distressful course of the condition, CPP is frequently managed within the biopsychosocial model of chronic pain—an approach, which emphasizes the importance of wholistic management of chronic pain conditions “pain itself along with associated psychological and social problems” for more positive treatment outcomes^2^. However, CPP women experience varying levels of psychological distress. Greater comorbidity (e.g., depression, poor sleep, fatigue, somatic symptoms) is more common among women who experience higher distress than those with little or no distress^16^. Therefore, careful identification of highly distressed women with CPP through the assessment of psychopathological symptoms is extremely pivotal for designing effective interventions for CPP and evaluating the outcomes of such interventions^2^.

The Depression, Anxiety, and Stress Scale-21 (DASS-21) is a simple measure frequently used in research and clinical practice to capture the distinct features of depression, anxiety, and stress symptomatology^17^. However, numerous studies reported enormous flaws associated with the DASS-21: variations in its dimensional structure^18–22^, non-invariance across different groups^23–25^, and a ceiling effect^26^. As a result, many revisions of its item structure took place.

An 18-item DASS was reported to express good fit in Asian samples from Malaysia, Indonesia, Singapore, Sri Lanka, Taiwan, and Thailand^27^. Another short version, DASS-14, was produced in an Australian sample of rehabilitation medical professionals based on the removal of items, which expressed poor loadings or loaded onto more than one factor^28^. These revised versions had a three-factor structure same as the parent scale, albeit with smaller inter-factor correlations^27^. New shorter forms were reported in other studies^22,29^.

Using several non-clinical samples, Osman et al. reported that the DASS-21 may be best used as a unidimensional measure of psychological distress^22^—a state of emotional suffering, which combines non-specific symptoms of depression and anxiety^30,31^. They also reported that numerous items poorly correlated with the underlying latent construct covered by the DASS-21, noting that reducing its items to 13 or nine items would remedy such flaws^22^. Subsequently, a 12-item version of the DASS-21 has been tested in non-clinical and clinical Korean samples^29^. However, this version has not been tested in another population until recently. A current study used clinical and non-clinical samples from Saudi Arabia to investigate various models of the parent scale as well as all the available shortened versions^32^. The fit of different models of the DASS-21 was considerably lower than all its short versions. An extensive revision of the item structure of the DASS-21 based on statistics and conceptual methods resulted in an 8-item version. The DASS-8 expressed excellent psychometric properties compared with Osman’s 13-item/nine-item DASS and the Korean DASS-12^32^. Likewise, in another investigation involving healthy individuals from the US, Australia, and Ghana, the DASS-8 expressed a better fit than the DASS-12. Examinations of discriminant validity revealed minimal overlapping of its subscales compared with the DASS-21 and the DASS-12. Its convergent validity, in terms of item loadings and item total correlations, was considerably better than the DASS-12. It also demonstrated more robust criterion validity by correlating with measures of internet addiction, adult attention-deficit/hyperactivity disorder (ADHD), and individualistic cultural orientation at similar or greater levels of significance^31^. In another investigation involving three Arab samples (individuals from COVID-19 quarantine facilities, psychiatric patients, and healthy adults), the DASS-8 and all its subscales significantly (all values < 0.01) correlated with COVID-19 trauma and its different dimensions: intrusion, avoidance, hyperarousal, numbing, sleep disturbance, and dysphoria^33^. Thus, the DASS-8 maybe reliably used as a criterion variable to detect debilitating morbidities (e.g., internet addiction, ADHD, and post-traumatic stress disorder (PTSD)), which are less frequently reported by patients and less recognized in primary care settings^31,33^.

Because they maximize response rates, brief screening instruments are intended to be used more frequently as clinical tools to facilitate the identification of pathological cases and enable assessing response to treatment^34,35^. Thus, there is an intense need to ensure the relevance, local precision, and adequate sensitivity of these instrument^35^. Although the DASS-8 and DASS-12 were invariant across different groups in Arab samples^32^, both short scales exhibited variance at the scalar level across English-speaking and Ghanaian participants, with the latter expressing lower levels of distress^31^. Individuals’ responses to the items of distress scales, such as the DASS-8, in different cultures may vary since individuals tend to selectively express their distress symptoms through culturally acceptable ways^36^. Therefore, to benefit from the DASS-8 as an available brief form of the DASS-21, it needs to be evaluated in more diverse populations, including patient groups, to ensure the adequacy of its sensitivity. Accordingly, this study aimed to examine the psychometrics of the DASS-8 in a sample of Australian women with CPP. We hypothesized that (1) the DASS-8 would express better fit and less non-variance than the DASS-12; (2) the DASS-8 would discriminate CPP women experiencing mental comorbidities from those without mental comorbidities; (3) the DASS-8 would correlate with the DASS-21 at the same level expressed by the DASS-12; (4) the DASS-8 would correlate with pain symptoms and history of sexual assault at the same level expressed by the parent scale; and (5) the DASS-8 may classify CPP women into classes of high and low levels of distress and morbidity. Thus, this paper complements existing knowledge in the field by expanding the methods used to examine the properties of the DASS-8, such as cluster analysis. Examinations of its criterion validity have been extended to aspects not addressed in prior research such as pain, sexual abuse, and collateral physical symptoms. Such criteria represent a source of enormous discomfort in CPP^3,8,11,37^.

## Material and methods

### Study design, participants, and procedure

This study is a secondary analysis based on a publicly accessible dataset from a previously published cross-sectional study^3^, which comprises a convenient sample of women with CPP. Data were collected via a pre-treatment self-administered questionnaire addressed to all clients attending a specialist pelvic pain clinic in South Australia over 18 months between January 2015 and July 2016. Women not signing informed consent, with several incomplete sections of the questionnaire, or solely experiencing period pain or dyspareunia were excluded from the study. Women or their guardian, if they were less than 18 years old, signed an informed consent. Because the protocol for data collection was previously approved by University of South Australia Human Research Ethics Committee (Application ID: 0000036598; 26/05/2017)^3^, and the dataset was publicly accessible^38^, we did not obtain an ethical approval for the current study.

### Measures

The questionnaire used for data collection comprised a large set of questions about pain experienced, its intensity, duration, pain-free days per month, pain severity during sexual activity, severity of stabbing pelvic pain, pain severity on the day of data collection, experiencing (tiredness/fatigue, anxiety, low mood, bad headache), somatic symptoms (e.g., nausea, unusual sweating, dizziness, and bloating), history of sexual assault, current psychiatric disorders, etc. Questions addressing pain severity or intensity prompted the respondents to rate the intensity of pain on a scale from 0 (no pain) to 10 (extremely severe pain)^3^.

The Depression Anxiety Stress Scale (DASS-21) was used to measure psychological distress. It comprises 21 items, in three subscales, which assess the symptoms of depression, anxiety and stress. Item responses are rated on a four-point scale ranging from 0 (did not apply to me at all) to 3 (applied to me very much or most of the time). The minimum and maximum total scores of the DASS-21 are 0 and 63^39^. The DASS-8 comprises eight items, in three subscales: depression (three items e.g., felt down hearted and blue), anxiety (three items e.g., felt scared without reason), and stress (two items e.g., was using a lot of my mental energy)^31,32^. The total scores of the DASS-8 and its subscales range between 0 to 24, 0 to 9, 0 to 9, and 0 to 6, respectively. The DASS-12 comprises 12 items, in three subscales, each comprising four items. The total scores of the DASS-12 and its subscales range between 0 to 24 and 0 to 12, respectively^29^. The reliability of the DASS-21, DASS-8, and DASS-12 in this sample is excellent (coefficient alpha = 0.95, 0.90, and 0.90, respectively). The inclusion of the DASS-21/DASS-12 in this study aims to ensure adequate validity of the DASS-8 as the shortest version of the DASS-21 relative to the parent scale and the DASS-12 as another longer brief version.

### Statistical analysis

First, we checked the dataset for missing responses. Because multiple items of the DASS-21 had missing responses, we removed all participants with incomplete data on the DASS-8 and DASS-12, which resulted in a final sample size of 214 participants—response rate = 90%. Meanwhile, 212 respondents were included in tests of the factor structure of the DASS-21. The normality of different versions of the DASS were tested by Shapiro Wilk W test. The statistics of non-normal variables are reported as median (MD) and interquartile range (IQR; Q1-Q3) while mean and standard deviation were used to report normally distributed variables. Number and percentage were used to describe categorical variables.

Based on two former investigations^31,32^, the factor structures of the DASS-8 and DASS-12 were examined using confirmatory factor analysis (CFA). To evaluate model fit as good or acceptable, we used chi square (χ^2^) index, ideally it should be non-significant, Comparative Fit Index (CFI) and Tucker–Lewis Index (TLI) equal to or above 0.95 and 0.90, respectively, in addition to standardized root-mean-square residual (SRMR) and root mean square error of approximation (RMSEA) less than 0.06 and 0.08, respectively^40^. This combination allows parsimonious evaluation of model fit because χ^2^ and RMSEA may be influenced by sample size, which if used alone may disqualify well-fitting models that express minor misspecifications^41,42^. Based on suggestions pointed by modification indices, few error terms were correlated to improve model fit.

To test measurement invariance of the DASS-8, age was categorized twice into two different groups: based on median age (110 women aged 31 years or below and 104 women above the age of 31) and based on the age reported to coincide with menopause transition (156 below the age of 40 versus 58 aged 40 years or above). This is because hormonal alterations in the perimenopause stage are associated with greater mood dysfunction than in the later post-menopause stage^7^. Then, multigroup CFA was conducted to compare invariance of the DASS-8 and DASS-12 at the configural, metric, scalar, and strict levels^43,44^ across groups of age, menopausal status (menopausal vs premenopausal), and psychiatric comorbidity (presence of comorbidity vs no comorbidity, the number of subjects in all categories are shown in Table 1). Significant changes in *χ*^2^ in constrained models, along with ΔCFI and ΔRMSEA above 0.02 and 0.015, respectively were used as criteria for non-invariance^30,43^.

**Table 1:** Sociodemographic and clinical characteristics of the participants.

Participant characteristics	Whole sample (N = 214)	Cluster 1 (n = 141)	Cluster 2 (n = 73)	p
Age in years mean (SD)	33.3 ± 12.4	33.8 ± 12.0	32.4 ± 13.4	0.477
Pain days mean (SD)	17.8 ± 9.6	16.9 ± 9.7	20.1 ± 9.1	0.014
Pain free days MD (IQR)	10.0 (2.8–20.0)	15.0 (4.0–21.0)	7.0 (1.0–15.0)	0.017
**Stabbing pelvic pain**				
Yes	185 (87.7%)	119 (85.6%)	66 (91.7%)	0.205
No	26 (12.3%)	20 (14.4%)	6 (8.3%)	
Stabbing pelvic pain severity mean (SD)	7.6 ± 1.8	7.3 ± 1.8	8.0 ± 1.7	0.015
Current pelvic pain severity MD (IQR)	4.0 (1.0–5.0)	3.0 (0–5.0)	5.0 (2.0–7.0)	0.001
**Sex pain**				
Yes	149 (82.8%)	100 (80.6%)	49 (87.5%)	0.259
No	31 (17.2%)	24 (19.4%)	7 (12.5%)	
Sex pain severity mean (SD)	6.0 ± 2.0	6.1 ± 2.0	7.3 ± 1.7	0.001
**Sexual assault**				
Yes	27 (16.1%)	8 (7.2%)	19 (33.3%)	0.001
No	141 (83.9%)	103 (92.8%)	38 (66.7%)	
Headache severity mean (SD)	7.0 ± 1.8	6.6 ± 1.9	7.7 ± 1.5	0.001
**Menopausal status**				
Premenopausal	188 (89.5%)	123 (88.5%)	65 (89.0%)	0.764
Menopausal	24 (11.4%)	16 (11.5%)	8 (11.0%)	
**Current psychiatric comorbidity**				
Yes	61 (29.3%)	26 (18.8%)	35 (50.0%)	0.001
No	147 (70.7%)	112 (81.2%)	35 (50.0%)	
**Comorbid psychiatric disorders**				
MDD	13 (21.3%)	7 (26.9%)	6 (17.1%)	0.774
GAD	14 (23.0%)	6 (23.1%)	8 (22.9%)	
MDD comorbid with GAD	28 (45.9%)	11 (42.3%)	17 (48.6%)	
Others	6 (9.8%)	2 (7.7%)	4(11.4%)	
**Bloating**				
Yes	163 (88.6%)	103 (85.8%)	60 (93.8%)	0.108
No	21 (11.4%)	17 (14.2%)	4 (6.2%)	
**Bowel pain**				
Yes	144 (78.7%)	86 (72.9%)	58 (89.2%)	0.010
No	39 (21.3%)	32 (27.1%)	7 (10.8%)	
**Subjective experience of anxiety**				
Yes	118 (58.1%)	52 (39.4%)	66 (93.0%)	0.001
No	39 (41.9%)	80 (60.6 ±)	5 (7.0%)	
**Subjective experience of low mood**				
Yes	118 (63.4%)	55 (47.0%)	63 (91.3%)	0.001
No	68 (36.6%)	62 (53.0%)	6 (8.7%)	
**Fatigue**				
Yes	146 (77.7%)	81 (68.1%)	65 (94.2%)	0.001
No	42 (22.3%)	38 (31.9%)	4 (5.8%)	
**Sleep problems**				
Yes	117 (59.4%)	63 (49.6%)	54 (77.1%)	0.001
No	80 (40.6%)	64(50.4%)	16(22.9%)	
**Dizziness**				
Yes	113 (58.9%)	60 (49.6%)	53 (77.9%)	0.006
No	79 (41.1%)	61(50.4%)	15(22.1%)	
**Unusual sweating**				
Yes	69 (34.8%)	35 (26.9%)	34 (50.0%)	0.002
No	129 (65.2%)	95 (73.1%)	34 (50.0%)	
**Nausea**				
Yes	92 (52.9%)	49 (33.6%)	43 (74.1%)	0.001
No	82 (47.1%)	97 (66.4%)	15 (25.9%)	

N ranges between 127 and 211, SD: standard deviation, MD: median, IQR: interquartile range, MDD: Major depression disorder, GAD: Generalized anxiety disorder.

To examine known-group validity of the DASS-21, DASS-12, DASS-8, Mann Whitney U test was used to determine whether these measures and their subscales can differentiate CPP women with comorbid psychiatric disorders from those without psychopathology. It was also used to differentiate women with depressive disorder from those with anxiety disorder. For discriminant validity, heterotrait-monotrait (HTMT) ratio of correlations of items comprising the DASS-21, DASS-12, DASS-8 were computed^31,45^. Moreover, two-step cluster analysis was used to determine whether the participants can be grouped according to the scores of the DASS-8 and its subscales. Clustering is a technique, which uses uncovered characteristics in a dataset to divide cases or variables into non-overlapping groups, with a high degree of similarity within each group and a low degree of similarity between groups^46^. Two-step cluster analysis is a hybrid technique that operates via two steps. The first step (pre-clustering) employs a sequential approach to separate groups by pre-clustering cases based on a distance measure that defines dense regions in the analyzed attribute-space. In the second step (clustering), a probabilistic approach is used to statistically merge pre-clusters stepwise until the optimal subgroup model is determined. This technique is highly reliable; in terms of the number of subgroups detected, classification probability of individuals into subgroups, and reproducibility of the findings on different types of data. It has additional merits: analyzing atypical values (i.e., outliers), determining the number of clusters based on a statistical measure of fit rather than on an arbitrary choice, using categorical and continuous variables simultaneously, and handling large datasets^47^. Independent sample t-test, Mann Whitney test, and *χ*^2^ were used to compare the differences in mental symptomatology and characteristics of the participants across clusters.

The reliability of the DASS-21, DASS-12, DASS-8, and their subscales was assessed by coefficient alpha, alpha-if-item deleted, and item-total correlations. The predictive validity of the DASS-12, DASS-8, and their subscales was detected by Spearman’s r correlating these measures to the original scale and its subscales. This test was also used to evaluate the criterion validity of the three measures by correlating their scores with sexual assault experience, number of pain days/pain-free days per month, pain severity during sexual activity, pain severity on the day of data collection, severity of stabbing pelvic pain, experiencing bad headache, experiencing tiredness/fatigue, experiencing anxiety, and experiencing low mood. All analyses were conducted in SPSS version 24, and significance was considered at a probability level less than 0.05 in two-tailed tests.

### Ethics approval and consent to participate

The protocol of data collection was approved by University of South Australia Human Research Ethics Committee (Application ID: 0000036598; 26/05/2017). All participants or their guardians signed a written informed consent before data collection^3^. No ethical approval was obtained for the current study because the analysis is based on a publicly accessible dataset^38^. The present study was conducted according to the Declaration of Helsinki.

## Results

### Characteristics of the participants

Participants in this study (N = 214, mean age = 33.1 ± 12.4 years) were women complaining from CPP. The participants experienced several types of pain (stabbing pelvic pain, sex pain, bowel pain, and headache), somatic symptoms (e.g., bloating, nausea, dizziness, unusual sweating), along with symptoms of low mood, anxiety, and psychiatric comorbidity. Table 1 shows more information on the sociodemographic and clinical characteristics of the participants in the overall sample as well as differences in these variables between women groups of low distress and high distress.

### Results of confirmatory factor analysis and invariance analysis

**Figure 1:**
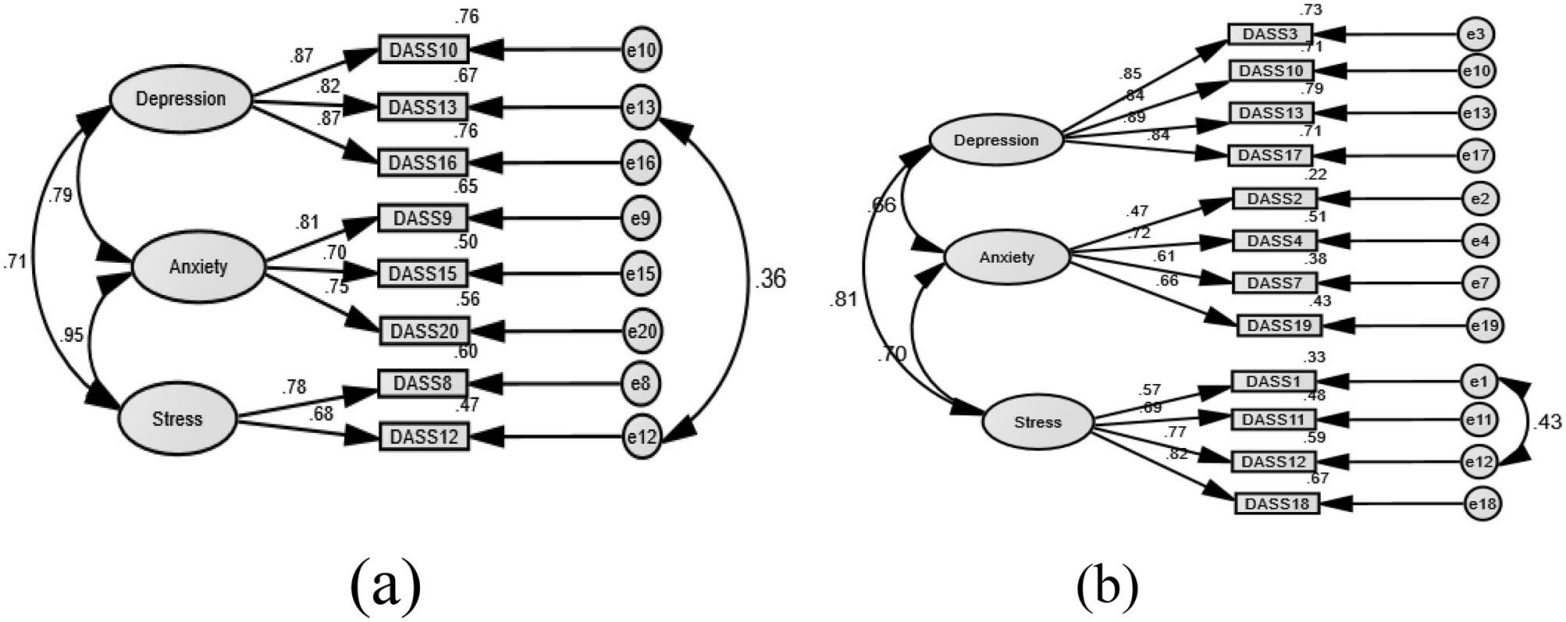
Factor structure of the short versions of the Depression Anxiety Stress Scale (DASS)-21: the DASS-8 (**a**) and the DASS-12 (**b**) among women with chronic pelvic pain.

As shown in Table 2, the one-factor structure of the DASS-8/DASS-12 expressed unsatisfactory fit. The crude models of the three-factor structures of the DASS-8 and the DASS-12 expressed good fit. However, RMSEA was on the high side for the DASS-8, suggesting minor misspecifications in item loadings, which may be overlooked given that all other fit indices indicated good fit (discussed in detail below). The fit of this model was considerably improved by correlating the residuals of item 12 and item 13. Likewise, the fit of the DASS-12 was slightly improved by correlating the residuals of item 1 and item 12 (Fig. 1b). On the other hand, the fit of all the crude models of the DASS-21 was poor. Correlating several error terms was necessary to produce an acceptable fit (Appendix 1: Fig. 1b). The fit of the second order structure was similar to that of the three-factor structure of all the DASS scales (Appendix 1, Appendix 2). While the bifactor structure of the DASS-8 expressed good fit with all items significantly loading on the general factor, item 12 and item 20 failed to load on their domain-specific factors of stress and anxiety, respectively (p > 0.05). In addition, item 15 loaded significantly on the anxiety factor (p = 0.04), but its loading on this factor was weak (β = 0.26). All the items of the anxiety subscale of the DASS-12 (Appendix 2: Supplementary Table 5) and the DASS-21 (Appendix 1: Table 2) failed to load on their corresponding factor in the bifactor model, and SRMR was not produced (Table 2, Appendix 1: Table 1), denoting failure of the model to converge adequately. Therefore, the three-factor structures of the DASS-8/DASS-12/DASS-21 represent the best fit for the data.

**Table 2:** Goodness-of-fit of the confirmatory factor analysis models representing the Depression Anxiety Stress Scale-8 (DASS-8) and the DASS-12 in the whole sample.

Models	Samples	χ^2^	*p*	*Df*	CFI	TLI	RMSEA	RMSEA 90% CI	SRMR
Model 1 1F DASS-8	Crude	154.108	0.001	20	0.869	0.817	0.171	0.146 to 0.198	0.0709
	Correlated error	106.028	0.001	19	0.909	0.866	0.147	0.120 to 174	0.0684
Model 2 3F DASS-8	Crude	51.086	0.001	17	0.964	0.941	0.097	0.067 to 0.128	0.0341
	Correlated error	31.261	0.012	16	0.984	0.972	0.067	0.030 to 0.102	0.0332
Model 3 bifactor DASS-8	Crude	30.999	0.013	16	0.984	0.973	0.066	0.030 to 0.101	0.0329
Model 4 1F DASS-12	Crude	268.420	0.001	54	0.840	0.805	0.137	0.121 to 0.153	0.0790
	Correlated error	176.317	0.001	52	0.907	0.882	0.106	0.089 to 0.123	0.0684
Model 5 3F DASS-12	Crude	117.403	0.001	51	0.951	0.936	0.078	0.060 to 0.097	0.0460
	Correlated error	88.049	0.001	50	0.972	0.963	0.060	0.038 to 0.080	0.0379
Model 6 bifactor DASS-12	Crude	113.763	0.001	51	0.953	0.937	0.078	0.060 to 0.097	–
	Correlated error	104.279	0.001	47	0.957	0.940	0.076	0.056 to 0.095	–

*χ*^2^, chi-square; df, degrees of freedom; CFI, comparative fit index; TLI, Tucker–Lewis index; RMSEA, root mean square error of approximation; CI, confidence interval; SRMR, standardized root mean residual; –, SRMR was not produced denoting improper convergence of the model.

As shown in Table 3, the three-factor structures of the DASS-8 and DASS-12 were invariant at the configural, metric, scalar, and strict levels across groups of age and menopausal status. For median-based age groups, there was a tendency toward non-invariance of the DASS-8 at the scalar level (ΔCFI > 0.02); however, ΔRMSEA was still within the acceptable range < 0.15. Obviously, the DASS-8 was non-invariant at the scalar level across groups of psychiatric comorbidity (ΔCFI > 0.02 and ΔRMSEA > 0.15). The DASS-12 was non-invariant at the strict level (ΔCFI > 0.02 and ΔRMSEA > 0.15), and it also tended to be non-invariant at the scalar level across comorbidity groups (SRMR = 0.1117).

**Table 3:** Invariance of the three-factor structures of the Depression Anxiety Stress Scale 8 (DASS-8) and DASS-12 across groups of age, menopausal status, and psychiatric comorbidity.

Model	Groups	Invariance levels	χ^2^	df	*P*	Δχ^2^	Δdf	*p*(Δχ^2^)	CFI	ΔCFI	TLI	ΔTLI	RMSEA	ΔRMSEA	SRMR
DASS-8	Age (≤ 31, > 31 years)	Configural	61.939	32	0.001				0.969	–	0.946	–	0.066		0.0382
		Metric	64.824	37	0.003	2.884	5	0.718	0.971	0.002	0.957	0.011	0.060	0.006	0.0373
		Strong	91.766	43	0.001	26.942	6	0.001	0.950	**0.021**	0.935	**0.022**	0.073	− 0.013	0.0680
		Strict	100.302	52	0.001	8.537	9	0.481	0.950	0.000	0.947	0.012	0.066	0.007	0.0669
DASS-12	Age (≤ 31, > 31 years)	Configural	164.318	100	0.001				0.954		0.939	–	0.055		0.0459
		Metric	175.896	109	0.001	11.579	9	0.238	0.952	0.002	0.941	0.002	0.054	0.001	0.0516
		Strong	201.610	115	0.001	25.714	6	0.001	0.937	0.015	0.928	0.013	0.060	− 0.006	0.0818
		Strict	248.104	128	0.001	46.494	13	0.001	0.913	**0.024**	0.911	0.017	0.067	− 0.07	0.0764
DASS-8	Age (< 40, ≥ 40 years)	Configural	47.004	32	0.042				0.984	–	0.973	–	0.047		0.0349
		Metric	50.139	37	0.073	3.136	5	0.679	0.986	0.002	0.979	0.006	0.041	0.006	0.0348
		Strong	60.908	43	0.037	10.769	6	0.096	0.981	0.005	0.976	0.003	0.044	− 0.003	0.0371
		Strict	73.699	52	0.026	12.791	9	0.172	0.977	0.004	0.976	0.000	0.044	0.000	0.0407
DASS-12	Age (< 40, ≥ 40 years)	Configural	164.205	100	0.001				0.954		0.940		0.055		0.0455
		Metric	188.199	109	0.001	23.994	9	0.004	0.944	0.010	0.932	0.008	0.059	− 0.004	0.0465
		Strong	201.257	115	0.001	13.058	6	0.042	0.939	0.005	0.930	0.002	0.059	0.000	0.0556
		Strict	244.418	128	0.001	43.161	13	0.001	0.917	**0.022**	0.915	**0.015**	0.065	− 0.006	0.0545
DASS-8	Menopausal status	Configural	52.020	32	0.014				0.979	0.011	0.964		0.055		0.0353
		Metric	67.719	37	0.002	15.699	5	0.008	0.968	0.008	0.952	0.012	0.063	0.008	0.0341
		Strong	81.087	43	0.001	13.369	6	0.038	0.960	–	0.949	0.003	0.065	− 0.002	0.0375
		Strict	89.725	52	0.001	8.638	9	0.471	0.961	0.001	0.958	0.009	0.059	0.006	0.0376
DASS-12	Menopausal status	Configural	207.346	100	0.001				0.924	–	0.899	–	0.071		0.0406
		Metric	213.566	109	0.001	6.220	9	0.718	0.926	0.002	0.910	0.011	0.068	0.003	0.0412
		Strong	219.012	115	0.001	5.446	6	0.488	0.926	0.000	0.915	0.005	0.066	0.002	0.0429
		Strict	249.779	128	0.001	30.767	13	0.767	0.913	0.013	0.911	0.003	0.067	− 0.001	0.0448
DASS-8	Psychiatric comorbidity	Configural	61.049	32	0.014				0.960		0.931		0.066		0.0598
		Metric	75.127	37	0.002	14.078	5	0.015	0.948	0.012	0.921	0.010	0.071	− 0.005	0.0677
		Strong	114.929	43	0.001	39.803	6	0.001	0.902	**0.046**	0.872	**0.049**	0.090	**0.019**	**0.1384**
		Strict	140.861	52	0.001	25.932	9	0.002	0.879	**0.023**	0.870	0.002	0.091	− 0.001	**0.0964**
DASS-12	Psychiatric comorbidity	Configural	161.746	100	0.001				0.943		0.925		0.055		0.0798
		Metric	175.878	109	0.001	14.132	9	0.118	0.938	0.005	0.925	0.000	0.055	0.000	0.0814
		Strong	198.757	115	0.001	22.879	6	0.001	0.923	0.016	0.911	0.014	0.059	− 0.004	**0.1117**
		Strict	268.320	128	0.001	69.562	13	0.001	0.871	**0.052**	0.867	**0.044**	0.073	− 0.014	**0.0965**

*χ*^2^, chi-square; df, degrees of freedom; CFI, comparative fit index; TLI, Tucker–Lewis index; RMSEA, root mean square error of approximation; CI, confidence interval; SRMR, standardized root mean residual.

Significant values are given in bold.

### Results of known-group validity and discriminant validity tests

As noted in Table 4, the DASS-21, DASS-8, DASS-12 differentiated women with a current psychiatric disorder from those without psychiatric comorbidity at the same level of significance (p < 0.001), which supports known-group validity of these measures. However, the z scores of the DASS-8 and its subscales were higher than those of the DASS-12 and the DASS-21, indicating a higher level of discriminant validity. Nonetheless, neither the DASS-21, DASS-8, DASS-12 nor their subscales could differentiate women with a diagnosis of depression from those with a diagnosis of anxiety. Likewise, the DASS-21, DASS-8, DASS-12, and their subscales significantly correlated with subjective experience of anxiety and low mood at the same levels (all p values < 0.01, Table 5). HTMT ratio of correlations showed that the constructs covered by the subscales of the DASS-8 and DASS-12 were distinct (< 0.85). However, the stress and anxiety subscales on the DASS-8 were overlapping (HTMT ratio = 0.95, Appendix 2: Supplementary Table 11). All the subscales of the parent scale exhibited overlap, albeit it was marginal for the stress-anxiety subscales (HTMT ratio = 0.85, Appendix 2: Supplementary Table 15).

**Table 4:** Descriptive statistics and discriminant validity of the Depression Anxiety Stress Scale 21 and its shortened versions among women with chronic pelvic pain.

DASS versions	Whole sample (N = 214)	Mann Whitney test	z	Cluster 1 (n = 141)	Cluster 2 (n = 73)	Mann Whitney test	z
	MD (IQR)			MD (IQR)	MD (IQR)		
DASS-21	11.5 (6.0–23.8)	2247.5	− 5.49	7.0 (4.0–11.8)	29.5 (23.0–36.8)	210.0	− 11.43
Depression	2.0 (1.0–7.0)	2495.5	− 5.08	1.0 (0–3.0)	9.0 (5.0–14.0)	1041.0	− 9.65
Anxiety	3.0 (1.0–7.0)	2482.0	− 5.11	1.0 (0–3.0)	8.0 (6.0–11.0)	728.0	− 10.39
Stress	6.0 (3.0–11.0)	2641.0	− 4.48	4.0 (2.0–6.0)	12.0 (10.0–16.0)	436.5	− 10.91
DASS-12	7.0 (3.0–13.3)	2320.0	− 5.48	4.0 (2.0–7.0)	17.0 (13.0–21.0)	397.0	− 11.08
Depression	1.0 (1.0–4.0)	2266.5	− 5.80	0 (0–1.0)	5.0 (3.0–8.5)	1082.0	− 9.77
Anxiety	2.0 (0–4.0)	2842.0	− 4.24	1.0 (0–2.0)	4.0 (3.0–6.0)	1514.5	− 8.63
Stress	4.0 (2.0–7.0)	2849.5	− 4.16	2.0 (1.0–4.0)	8.0 (6.0–9.5)	856.0	− 10.05
DASS-8	4.0 (1.0–8.0)	2018.0	− 6.27	2.0 (1.0–3.0)	12.0 (8.0–15.0)	36.0	− 11.95
Depression	1.0 (0–3.0)	2346.5	− 5.60	0 (0–1.0)	4.0 (2.0–6.0)	1214.0	− 9.47
Anxiety	1.0 (0–3.0)	2521.0	− 5.21	0 (0–1.0)	4.0 (3.0–6.0)	674.0	− 10.93
Stress	2.0 (1.0–3.0)	2447.0	− 5.26	1.0 (0–2.0)	4.0 (3.0–5.0)	364.0	− 11.37

DASS: Depression Anxiety Stress Scale, MD: median, IQR: interquartile range, U: Mann Whitney U test, p values were all significant at 0.001 level. For the DASS-21 (N = 212).

**Table 5:** Internal consistency, predictive validity, normality, and criterion validity of the Depression Anxiety Stress Scale (DASS) 21, DASS-12, DASS-8, and their subscales among women with chronic pelvic pain.

Criteria	DASS-21	Depression	Anxiety	Stress	DASS-12	Depression	Anxiety	Stress	DASS-8	Depression	Anxiety	Stress
Coefficient alpha	0.945	0.931	0.837	0.878	0.898	0.845	0.693	0.827	0.901	0.892	0.794	0.705
Range of corrected item-total correlations	0.401–0.776	0.598–0.847	0.370–0.713	0.593–0.735	0.401–0.785	0.607–0.719	0.371–0.550	0.607–0.746	0.629–0.744	0.882–0.944	0.634–0.662	0.545
Range of alpha if-item-deleted	0.940–0.946	0.914–0.939	0.795–0.853	0.851–0.869	0.881–0.902	0.787–0.833	0.585–0.708	0.742–0.809	0.884–0.893	0.831–0.870	0.702–0.733	–
Correlation with subscale of the DASS-21	–	–	–	–	–	0.930***	0.919***	0.948***	–	0.934***	0.871***	0.871***
Correlation with the DASS-21	–	0.856***	0.823***	0.929***	0.966***	0.838***	0.718***	0.863***	0.943***	0.811***	0.789***	0.818***
Shapiro Wilk test	0.892***	0.812***	0.848***	0.936***	0.906***	0.787***	0.843***	0.933***	0.864***	0.788***	0.790***	0.891***
Correlation with sexual assault experience	0.203**	0.264***	0.196**	0.134	0.192*	0.278***	0.095	0.106	0.259**	0.245**	0.261***	0.188*
Correlation with pain days/month	0.183**	0.137	0.221**	0.183**	0.193**	0.190**	0.204**	0.136	0.180*	0.177*	0.172*	0.163*
Correlation with pain-free days/month	− 0.182**	− 0.132	− 0.217**	− 0.174*	− 0.193**	− 0.188**	− 0.201**	− 0.139*	− 0.179*	− 0.173*	− 0.168*	− 0.168*
Correlation with severity of pain today	0.265***	0.217**	0.331***	0.238**	0.290***	0.255***	0.291***	0.239**	0.265***	0.225**	0.274***	0.234**
Correlation with severity of stabbing pelvic pain	0.200**	0.167*	0.287**	0.138	0.209**	0.174*	0.246**	0.143	0.205**	0.149*	0.246**	0.153*
Correlation with severity of pain during sexual activity	0.262**	0.308***	0.210*	0.222**	0.280**	0.326***	0.187*	0.229**	0.257**	0.285**	0.218**	0.227**
Correlation with severity of bad headache	0.387***	0.300**	0.253**	0.378***	0.395***	0.324***	0.221*	0.363***	0.299**	0.303**	0.225*	0.234**
Correlation with experienced anxiety	0.517**	0.430**	0.412**	0.491**	0.498**	0.427**	0.323**	0.478**	0.531**	0.425**	0.473**	0.467**
Correlation with experienced low mood	0.487**	0.482**	0.323**	0.446**	0.478**	0.512**	0.227**	0.447**	0.514**	0.492**	0.400**	0.412**
Correlation with experienced fatigue	0.345**	0.296**	0.333**	0.305**	0.348**	0.300**	0.346**	0.288**	0.322**	0.283**	0.231**	0.298**
Correlation with poor sleep	0.303**	0.250**	0.337**	0.248**	0.305**	0.227**	0.329**	0.238**	0.283**	0.246**	0.240**	0.257**
Correlation with dizziness	0.251**	0.174**	0.333**	0.191**	0.243**	0.192**	0.335**	0.150*	0.244**	0.181**	0.262**	0.211**
Correlation with unusual sweating	0.238**	0.182**	0.280**	0.215**	0.239**	0.174*	0.306**	0.197**	0.222**	0.179**	0.189**	0.257**
Correlation with nausea	0.256**	0.240**	0.339**	0.180*	0.259**	0.221**	0.349**	0.145*	0.236**	0.219**	0.260**	0.154*
Correlation with bloating	0.259**	0.216**	0.255**	0.206**	0.247**	0.213**	0.234**	0.193**	0.279**	0.245**	0.234**	0.246**

*, **, ***: correlation is significant at a level of 0.05, 0.01, 0.001, respectively.

In two-step cluster analysis, the DASS-8 and its subscales classified the participants into two clusters: low distress (cluster 1: n = 141, 65.9%) and high distress (cluster 2: n = 73, 34.1%). The model expressed good fit as indicated by Silhouette measure of cohesion and separation of around 0.7 and ratio of sizes less than 3 (1.93). Values of the predictor importance of the DASS-8 followed by stress, anxiety, and depression were 1, 0.85, 0.73, and 0.5, respectively. Mann Whitney U test revealed significant differences in the level of all mental distress symptoms among participants in both clusters—they were all significantly higher in cluster 2 than in cluster 1 (all p values < 0.001), with the DASS-8 and its stress subscale expressing the highest z scores (Table 4).

Age, menopausal status, bloating, and the frequency of stabbing pelvic pain and sex pain did not vary significantly across clusters. However, participants in cluster 2 demonstrated significantly higher number of pain days (t(146.9) = − 2.50, p = 0.014), less pain free days (U = 3654.0, z = − 2.39, p = 0.017); more severity of stabbing pelvic pain (t(134.42) = − 2.47, p = 0.015), sex pain (t(105.22) = − 3.74, p = 0.001), current pelvic pain (U = 3095.0, z = − 3.29, p = 0.001), concurrent headache (t(118.11) = − 3.51, p = 0.001); as well as higher frequency of bowel pain (*χ*^2^(1) = 6.68, p = 0.010), greater occurrence of sexual assault (*χ*^2^(1) = 19.06, p = 0.001), psychiatric co-morbidity (*χ*^2^(2) = 23.61, p = 0.001), subjective experience of psychiatric symptoms ((low mood (*χ*^2^(16) = 29.8, p = 0.001) and anxiety (*χ*^2^(1) = 50.50, p = 0.001)), fatigue (*χ*^2^(1) = 14.12, p = 0.001), and sleep problems (*χ*^2^(16) = 12.13, p = 0.001)), as well as somatic symptoms ((dizziness (*χ*^2^(1) = 7.57, p = 0.006), unusual sweating (*χ*^2^(1) = 9.52, p = 0.002), and nausea (*χ*^2^(1) = 13.65, p = 0.001)).

### Results of tests of reliability, normality, and criterion validity

The reliability of the DASS-21, DASS-8, and DASS-12 was excellent. Meanwhile, the reliability of the shortened subscales ranged from very good to poor (Table 5)—poor reliability was reported only for the anxiety subscale of the DASS-12. The predictive validity of the DASS-8, DASS-12, and their subscales was depicted by their strong correlation with the original scale and its subscales (Table 5). The normality of the DASS-8 and the DASS-12 was comparable with that of the DASS-21 as noted by Shapiro–Wilks’ W. As shown in Table 5, all the DASS versions and most of their subscales negatively correlated with pain-free days and positively correlated with pain experience on the survey day, pain days per month, concurrent headache, poor sleep, fatigue, somatic symptoms (nausea, bloating, dizziness, unusual sweating), experience of low mood and anxiety, as well as sexual assault experience. Notably, all the subscales of the DASS-8 had significant positive correlations with sexual assault experience while the anxiety and stress subscales of the DASS-12 as well as the stress subscale of the DASS-21 did not significantly correlate with this variable. Similarly, all the subscales of the DASS-8 significantly correlated with the severity of stabbing pelvic pain while the stress subscale of the DASS-12 and the DASS-21 failed to significantly correlate with this variable (Table 5).

## Discussion

Distress management may considerably affect CPP recovery^2^. This is because a considerable proportion of CPP patients experience excessive levels of distress^2,8,9,16^. Because resources allocated for disease management are limited^16^, there is a great need for brief measures that may facilitate the identification of highly distressed patients as well as their response to treatment^35^. Using numerous robust psychometric testing techniques, the present study suggests usefulness of the DASS-8 as a measure of distress, depression, anxiety, and stress symptomatology among Australian women with CPP.

The crude models of the three-factor structure of the DASS-8/DASS-12/DASS-21 expressed better fit than all other crude models. As for the DASS-8, the value of RMSEA was on high while other fit indices indicated good fit. In simple CFA and path models, which have few degrees of freedom (df), RMSEA may incorrectly indicate poor fit, even when data actually fit the model well^48,49^. However, other absolute fit indicators such as SRMR and CFI are less influenced by the effects of df in these models compared with RMSEA. The confidence intervals and p-values of SRMR and CFI are recorded to provide more accurate information on differentiating models with various levels of misfit^49^. Therefore, in models with small df, researchers are recommended to rely more on SRMR and CFI and take caution when interpreting RMSEA or completely avoid its use^49,50^. The value of RMSEA for the bifactor structure based on the three-factor model of the DASS-8 (Model 3, Table 2) was within the acceptable bound, without a need to correlate any errors, which suggests that RMSEA produced for the three-factor structure of the DASS-8 reflects a pseudo-misfit.

The fit of the three-factor structure of all the DASS scales was improved by correlating few error terms (Fig. 1, Appendix 1: Fig. 1b). The correlation involving item 12 “I found it difficult to relax” on the stress subscale and item 13 “I felt down-hearted and blue” on the depression subscale of the DASS-8 may suggest overlap of negative affect with comorbidities in this sample. This logic is based on the findings of network analyses comprising data from community-dwelling adults with a history of trauma: “inability to relax” and “diminished positive emotions” were key hub symptoms, which connected major distinct symptom groups that were identified as presentations of the disorders of depression, generalized anxiety, and PTSD, denoting psychopathological comorbidity in that sample^51^. In our study, item 12 and item 13 were strongly correlated (r = 0.58, p = 0.001). Moreover, the levels of symptoms expressed by both items, in order, were significantly higher among those with a formal current diagnosis of mental disorders (U = 2745.5, 2507.5; z = − 4.6 4, − 5.38; p values = 0.001) as well as those who reported sexual assault (U = 1315.5, 1037.5; z = − 2.684, − 4.03; p = 0.001 and 0.007) compared with disease- and abuse-free women. On the other hand, correlations involving item 12 and item 1 “I found it difficult to wind down” on the stress subscale of the DASS-12 (Fig. 1b) and the DASS-21 may obviously be related to the fact that the meanings of “difficult to relax” and “difficult to wind down” are almost the same—a possible indicator of item redundancy. In fact, several items of the stress subscale of the DASS-21 were removed in a former testing in numerous Asian samples, resulting in the revised DASS-18. This short version demonstrated a cleaner factorial structure and smaller inter-factor correlations relative to the DASS-21^27^.

The final model of the DASS-8 (Model 2 with correlated errors) expressed a perfect fit on all CFA fit indices, superior to the DASS-12, indicating a better construct validity of the DASS-8 as we hypothesized. Meanwhile, the fit of the second-order factor structure of all DASS scales was similar to the three-factor structure (Appendix 1, Appendix 2), indicating usability of the total score and the scores of the subscales of these scales. Evidently more error correlations were necessary to achieve an acceptable fit of the three-factor structure of the DASS-21 (Appendix 1: Fig. 1b). Of interest, correlated errors noticed in the structures of the DASS-8/DASS-12 were among those suggested to improve the fit of three-factor structure of the parent scale, which supports an interaction of comorbidities in this population and item redundancy as argued above.

The three-factor structures of both short scales exhibited configural, metric, scalar, and strict invariance across groups of age and menopausal status. Across groups of psychiatric comorbidity, the DASS-8 was non-invariant at the scalar level and the DASS-12 expressed misfit (indicating tendency toward non-invariance) at the scalar level and it was non-invariant at the strict level. Critical ratios for differences between parameters indicated that non-invariance of the DASS-8 involved: (1) lower loadings of item 20 “I felt scared without any good reason” on the anxiety subscale and item 12 on the stress subscale, (2) weaker correlation between the anxiety and depression subscales, and (3) less covariance between items 12 and 13. All were noted among participants not diagnosed with a mental disorder compared with those having a psychiatric disorder. This result is congruent with our aforementioned argument concerning a possible effect of comorbidity on the interactions taking place among the items and factors of the DASS-8. Non-invariance of the DASS-12 involved differences in the loading of item 17 “I felt I was not worth much as a person” and the variances of items 1, 3 “I couldn’t seem to experience any positive feeling at all”, and 10 “I felt that I have nothing to look forward”—all on the depression subscale.

The DASS-8 can be a beneficial brief measure for identifying CPP women with psychiatric comorbidity, high level of distress, and other debilitating symptoms (e.g., greater pain severity, poor sleep, concurrent headache, gastrointestinal discomfort, etc.). This is because the DASS-8 adequately differentiated CPP women with psychiatric comorbidities from those who are mental illness free. Although known-group validity of the DASS-8, DASS-12, and DASS-21 was expressed at the same level of significance (p = 0.001), the z scores associated with the DASS-8 were higher than those of the DASS-12 and DASS-21 (Table 4). This finding along with HTMT ratios denote superior discriminant validity of the DASS-8. This finding is consistent with a former investigation in which the DASS-8 potently identified psychiatric patients from healthy subjects^32^. On the other side, the DASS-8 as well as the DASS-12 and the DASS-21 could not discriminate CPP women with depression disorder from those with anxiety disorder. In line, the DASS-21 and the DASS-12 have been previously reported to lack the capacity to differentiate depression disorder from anxiety disorder^21,29,52^. These findings are consistent with other results on the reported comorbidity of both depression and anxiety disorders, confirming that the DASS measure is not a clinically diagnostic tool, but it can efficiently identify individuals prone to both depression and anxiety psychopathologies^53,54^. This can be a merit of a brief self-administered measure. Extra diagnostic workout can be performed in a next step to attain a formal diagnosis.

In two-step cluster analysis, the DASS-8 and its subscales classified CPP women into two clusters with low and high levels of distress. The DASS-8 and its stress subscale had higher predictor importance values than anxiety and depression. Mann Whitney U test revealed significant differences (all p values = 0.001) in the scores of all the DASS measures among women in both clusters, with the DASS-8 and its stress subscale exhibiting the highest z scores (Table 4). As shown in Table 1, participants in cluster 2 (high distress) scored significantly higher on symptoms of pain severity, bowel pain, depression, anxiety, fatigue, dizziness, nausea, and unusual sweating than participants in cluster 1. This finding is consistent with a former study, which classified women into two clusters: one cluster comprised women high on depression, fatigue, and poor sleep while the other cluster comprised women experiencing no or minimal levels of these symptoms^16^. However, Mann Whitney U test and cluster analysis in the present study indicate stronger discriminant validity of the DASS-8 and its stress and anxiety subscales than the depression subscale. Overall, the DASS-8 can be efficiently used to differentiate highly distressed CPP women (e.g., with psychiatric comorbidity and more severity of mental symptoms, pain, and physical symptoms) from those with low levels of distress.

Apart from good fit, invariance, and adequate discriminant validity, the DASS-8 demonstrates other excellent psychometric characteristics. As shown in Table 5, the internal consistency of the DASS-8 and all its subscale was good—considerably higher than that of the DASS-12 as we hypothesized, except for the stress subscale, which comprises half the number of items on the corresponding subscale of the DASS-12. Whereas the values of alpha-if-item deleted indicated reduction in the reliability of the DASS-8, they indicated an increase in the reliability of the DASS-12 and its anxiety subscale (Table 5), lending support to the more robust convergent validity of the DASS-8. Consistent with our preset hypothesis, strong correlations of the DASS-8 and its subscales with the DASS-21 and its subscales convey adequate item coverage, predictive validity, and convergent validity. Generally, these results highlight the high homogeneity, specificity, and sensitivity of the items comprising the DASS-8, which have been reported among Arab and English-speaking participants^31,32^.

In parallel, the DASS-8 and all its subscales exhibited significant correlations with sexual assault, pain duration (pain days per months), pain-free days, pain intensity, somatic symptoms, fatigue, poor sleep, bad headache, etc. On the other hand, the stress and depression subscales of the DASS-21 failed to significantly correlate with sexual assault and pain-free days, respectively. Likewise, the anxiety and depression subscales of the DASS-12 failed to significantly correlate with sexual assault while the correlation of the overall DASS-12 with sexual assault was at a lower level than that expressed by the DASS-8 (r = 0.192, p = 0.05 vs r = 0.259, p = 0.01). These findings indicate better criterion validity of the DASS-8. They are also congruent with recent studies reporting significant correlations of the subscales of the DASS-21 with stabbing pelvic pain, migraine, intimate partner violence/domestic violence; as well as early childhood physical, sexual and emotional abuse among women^3,14,15,55^. Therefore, the DASS-8 can be reliably used as a valid criterion to predict different noxious symptoms and experiences in CPP.

This study is the first attempt to confirm the psychometric soundness of the DASS-8 as a measure of mental symptomatology among Australian women with CPP. Results obtained from different robust testing techniques in this study emphasize excellent psychometric properties of the DASS-8; in terms of fit, invariance, predictive validity, convergent validity, discriminant validity, criterion validity, and reliability. These finding are all in line with those previously reported in Arab psychiatric patients as well as in healthy respondents from Saudi Arabia, Australia, the US, and Ghana^31,32^. In the meantime, this study is different from previous studies given the chronic and complicated nature of CPP. It extended the criterion validity of the DASS-8 by relating it to collateral physical complaints, pain variables, and history of sexual abuse—the latter was not correlated to one subscale of the DASS-21 and two subscales of the DASS-12. Another key originality aspect of the present study was revealed by cluster analysis—the DASS-8 classified CPP women into a low-distress group and a high-distress group. As shown in Tables 1 and 4, the high-distress group is evidently in needed for further diagnostic investigations and treatment because its members scored high on pain severity variables, several physical complaints, the DASS-21, and the DASS-12. Therefore, the DASS-8 can be used as a brief scale to identify CPP women who are prone to physical comorbidity and psychopathology, which may have implications for the diagnosis and treatment of psychiatric comorbidity among these women, eventually resulting in improved pelvic pain recovery.

This study has also many limitations, which should be acknowledged. The generalizability of the finding is limited out of possible risk for selection bias: (1) the sample is collected from a single clinic, (2) a priori power analysis for determining the sample size is lacking, (3) data on the respondents who declined participation in the survey are lacking, (4) the inclusion and exclusion criteria are not clearly defined, and (5) the results are based on a convenience sample, which may not represent all CPP women in other settings or countries. The cross-sectional design used precludes test–retest reliability testing. There is no information available on the validity of the measures of pain and physical complaints, which may cast doubt on the soundness of the relationships that are reported in criterion validity tests. Thus, future investigations may use a longitudinal design to examine the properties of the DASS-8 as well as causal direction of effects addressed in this study, along with mechanisms underlying links between psychological distress, pain, sleep problems, concurrent headache, etc. Moreover, all the published evaluations of the DASS-8 are produced by the same group of authors/co-authors, which may entail a risk of bias for reporting and interpreting the available results. Therefore, more investigations by independent researchers are needed.

## Conclusion

Psychometric evaluation of the DASS-8 among CPP women by numerous robust techniques revealed its proper fit, invariance, high reliability, good convergent validity, sound discriminant validity, adequate predictive validity, and good criterion validity. The results indicate usability of the DASS-8 as a brief, reliable, invariant measure of mental symptoms in reproductive age and menopausal women with CPP. Timely identification of highly distressed women would encourage prompt use of relevant psychiatric intervention. Owing to its brevity, the DASS-8 can facilitate frequent monitoring of common mental symptomatology over the course of CPP treatment, supporting efforts directed toward improving recovery in CPP population.

## Supplementary Information

Supplementary Legends.

Supplementary Information 2.

Supplementary Information 3.

## Data Availability

The dataset^38^ supporting the conclusions of this article is available in Zenodo repository, (https://zenodo.org/record/1307252#.YckoVWhBw2w), and also the datasets used and/or analyzed during the current study are available from the corresponding author on reasonable request.
