# Supplementary material for: Psychometric evaluation of the Depression Anxiety Stress Scale 8 among women with chronic non-cancer pelvic pain
Source: Sci Rep. 2022 Nov 30;12:20693. doi: 10.1038/s41598-022-15005-z (PMC9712382; doi:10.1038/s41598-022-15005-z)
Supplement: Supplementary file 1 — Supplementary Legends. [file 41598_2022_15005_MOESM1_ESM.docx]

**Appendix 1.** Factor analysis of the Depression Anxiety Stress 21 (DASS-21).

**Appendix 2.** Supplementary excel 1 showing extra relevant analyses that are not reported in the text.
