# Supplementary material for: Psychometric evaluation of the Depression Anxiety Stress Scale 8 among women with chronic non-cancer pelvic pain
Source: Sci Rep. 2022 Nov 30;12:20693. doi: 10.1038/s41598-022-15005-z (PMC9712382; doi:10.1038/s41598-022-15005-z)
Supplement: Supplementary file 2 — Supplementary Information 2. [file 41598_2022_15005_MOESM2_ESM.docx]

| 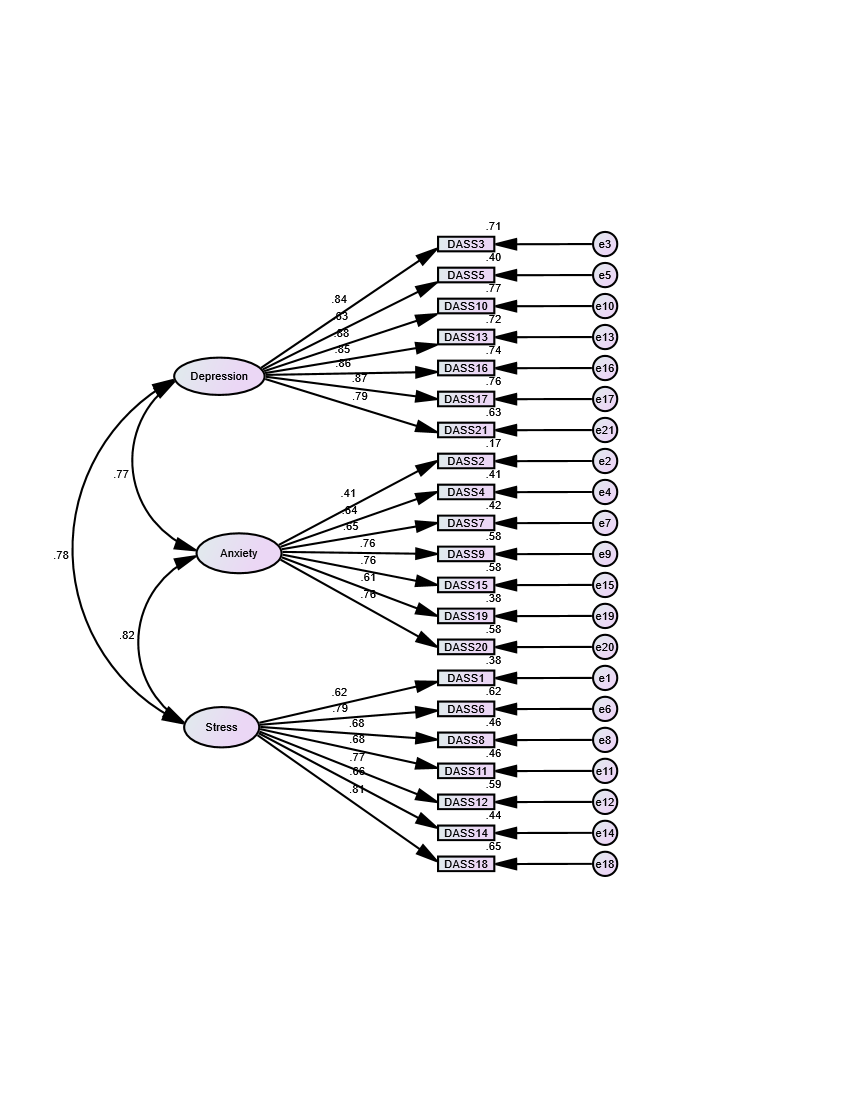  (a) | 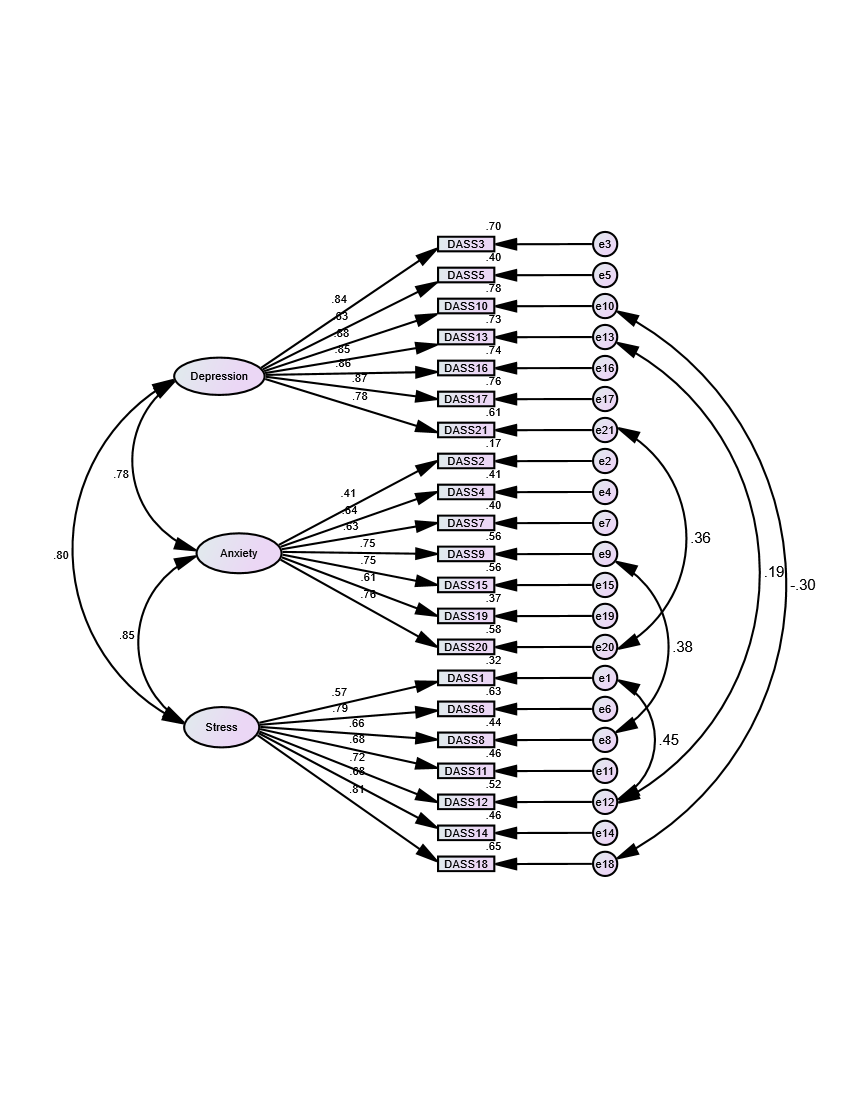  (b) |
| --- | --- |

Figure 1. Three-factor structure of the DASS-21 with no error correlation (a) and with correlated errors (b).

Table 1. Fit indices of different dimensional models of the DASS-21 tested by CFA

| **Models** | **Samples** | **χ^2^** | ***p*** | ***df*** | **CFI** | **TLI** | **RMSEA** | **RMSEA 90% CI** | **SRMR** |
| --- | --- | --- | --- | --- | --- | --- | --- | --- | --- |
| Model 1  1F DASS-8 | Crude | 810.169 | 0.001 | 189 | 0.786 | 0.762 | 0.125 | 0.116 to 0.134 | 0.0766 |
|  | Correlated error | 648.122 | 0.001 | 185 | 0.840 | 0.819 | 0.109 | 0.100 to 118 | 0.0703 |
| Model 2  3F DASS-21 | Crude | 515.740 | 0.001 | 186 | 0.886 | 0.872 | 0.092 | 0.082 to 0.101 | 0.0595 |
|  | Correlated error | 405.058 | 0.001 | 181 | 0.923 | 0.910 | 0.077 | 0.067 to 0.087 | 0.0522 |
| Model 3 bifactor DASS-21 | Crude | 585.402 | 0.001 | 184 | 0.896 | 0.882 | 0.088 | 0.079 to 0.098 | -- |
| Model 4  Second order DASS-21 | Crude | 515.740 | 0.001 | 186 | 0.886 | 0.872 | 0.092 | 0.082 to 0.101 | 0.0595 |
|  | Correlated error | 405.058 | 0.001 | 181 | 0.923 | 0.910 | 0.077 | 0.067 to 0.087 | 0.0522 |

**Abbreviations:** *χ* ^2^, chi-square; df, degrees of freedom; CFI, comparative fit index; TLI, Tucker–Lewis index; RMSEA, root mean square error of approximation; CI, confidence interval; SRMR, standardized root mean residual, --: SRMR was not produced for this model.

Table 2. Regression Weights corresponding to item loadings on their domain specific factor and the general factor in the bifactor structure of the DASS-21: (Group number 1 - Default model)

|  |  |  | Estimate | S.E. | C.R. | P | Label |
| --- | --- | --- | --- | --- | --- | --- | --- |
| DASS2 | <--- | Anxiety | 1.000 |  |  |  |  |
| DASS4 | <--- | Anxiety | 13.302 | 190.604 | .070 | .944 |  |
| DASS7 | <--- | Anxiety | 32.374 | 468.421 | .069 | .945 |  |
| DASS9 | <--- | Anxiety | -14.598 | 216.754 | -.067 | .946 |  |
| DASS15 | <--- | Anxiety | 18.354 | 264.142 | .069 | .945 |  |
| DASS19 | <--- | Anxiety | 28.129 | 406.936 | .069 | .945 |  |
| DASS20 | <--- | Anxiety | -20.718 | 305.845 | -.068 | .946 |  |
| DASS3 | <--- | Depression | 1.000 |  |  |  |  |
| DASS5 | <--- | Depression | .575 | .175 | 3.284 | .001 |  |
| DASS10 | <--- | Depression | 1.646 | .196 | 8.396 | *** |  |
| DASS13 | <--- | Depression | 1.207 | .163 | 7.415 | *** |  |
| DASS16 | <--- | Depression | 1.279 | .161 | 7.934 | *** |  |
| DASS17 | <--- | Depression | 1.376 | .171 | 8.026 | *** |  |
| DASS21 | <--- | Depression | 1.133 | .153 | 7.426 | *** |  |
| DASS1 | <--- | Stress | 1.000 |  |  |  |  |
| DASS6 | <--- | Stress | .720 | .152 | 4.746 | *** |  |
| DASS8 | <--- | Stress | .429 | .148 | 2.896 | .004 |  |
| DASS11 | <--- | Stress | 1.040 | .214 | 4.871 | *** |  |
| DASS12 | <--- | Stress | .982 | .180 | 5.448 | *** |  |
| DASS14 | <--- | Stress | .458 | .158 | 2.896 | .004 |  |
| DASS18 | <--- | Stress | .935 | .174 | 5.372 | *** |  |
| DASS3 | <--- | Distress | .596 | .033 | 18.264 | *** | a |
| DASS5 | <--- | Distress | .596 | .033 | 18.264 | *** | a |
| DASS10 | <--- | Distress | .596 | .033 | 18.264 | *** | a |
| DASS13 | <--- | Distress | .596 | .033 | 18.264 | *** | a |
| DASS16 | <--- | Distress | .596 | .033 | 18.264 | *** | a |
| DASS17 | <--- | Distress | .596 | .033 | 18.264 | *** | a |
| DASS21 | <--- | Distress | .596 | .033 | 18.264 | *** | a |
| DASS2 | <--- | Distress | .596 | .033 | 18.264 | *** | a |
| DASS4 | <--- | Distress | .596 | .033 | 18.264 | *** | a |
| DASS7 | <--- | Distress | .596 | .033 | 18.264 | *** | a |
| DASS9 | <--- | Distress | .596 | .033 | 18.264 | *** | a |
| DASS15 | <--- | Distress | .596 | .033 | 18.264 | *** | a |
| DASS19 | <--- | Distress | .568 | .057 | 9.919 | *** |  |
| DASS20 | <--- | Distress | .596 | .033 | 18.264 | *** | a |
| DASS1 | <--- | Distress | .596 | .033 | 18.264 | *** | a |
| DASS6 | <--- | Distress | .596 | .033 | 18.264 | *** | a |
| DASS8 | <--- | Distress | .596 | .033 | 18.264 | *** | a |
| DASS11 | <--- | Distress | .596 | .033 | 18.264 | *** | a |
| DASS12 | <--- | Distress | .596 | .033 | 18.264 | *** | a |
| DASS14 | <--- | Distress | .596 | .033 | 18.264 | *** | a |
| DASS18 | <--- | Distress | .596 | .033 | 18.264 | *** | a |
